# Challenges to Accurate Estimation of Methane Emission from Septic Tanks with Long Emptying Intervals

**DOI:** 10.1021/acs.est.3c05724

**Published:** 2023-10-19

**Authors:** Jakpong Moonkawin, Loi T. Huynh, Mariane Y. Schneider, Shigeo Fujii, Shinya Echigo, Lien P. H. Nguyen, Thu-Huong T. Hoang, Hai T. Huynh, Hidenori Harada

**Affiliations:** † Graduate School of Global Environmental Studies, Kyoto University, Kyoto 606-8501, Japan; ‡ Faculty of Environment, School of Technology, Van Lang University, Ho Chi Minh City 70000, Viet Nam; § Next Generation Artificial Intelligence Research Center & School of Information Science and Technology, The University of Tokyo, 113-8656 Tokyo, Japan; ∥ BIOMATH, Department of Data Analysis and Mathematical Modelling, Ghent University, Coupure Links 653, Ghent 9000, Belgium; ⊥ Centre for Advanced Process Technology for Urban Resource Recovery (CAPTURE), Frieda Saeysstraat 1, Gent 9000, Belgium; # School of Chemistry and Life Science, Hanoi University of Science and Technology, Hanoi 10000, Viet Nam; ¶ Graduate School of Asian and African Area Studies, Kyoto University, Kyoto 606-8501, Japan

**Keywords:** septic tanks, long emptying intervals, methane emission, pollutant removal, on-site sanitation, greenhouse gas, climate change mitigation

## Abstract

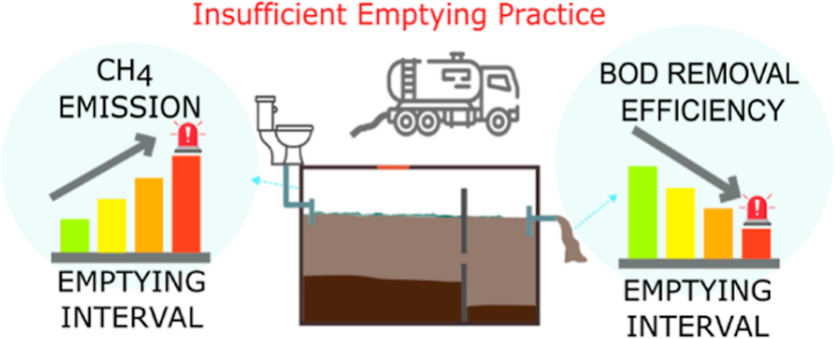

Septic tanks in low- and middle-income countries are often not emptied for a long time, potentially resulting in poor pollutant removal efficiency and increased greenhouse gas emissions, including methane (CH_4_). We examined the impact of long emptying intervals (4.0–23 years) on the biochemical oxygen demand (BOD) removal efficiency of 15 blackwater septic tanks and the CH_4_ emission rates of 23 blackwater septic tanks in Hanoi. The average BOD removal efficiency was 37% (−2–65%), and the average CH_4_ emission rate was 10.9 (2.2–26.8) g/(cap·d). The emptying intervals were strongly negatively correlated with BOD removal efficiency (*R* = −0.676, *p* = 0.006) and positively correlated with CH_4_ emission rates (*R* = 0.614, *p* = 0.001). CH_4_ emission rates were positively correlated with sludge depth (*R* = 0.596, *p* = 0.002), but against expectation, negatively correlated with BOD removal efficiency (*R* = −0.219, *p* = 0.451). These results suggest that shortening the emptying interval improves the BOD removal efficiency and reduces the CH_4_ emission rate. Moreover, the CH_4_ emission estimation of the Intergovernmental Panel on Climate Change, which is a positive conversion of BOD removal, might be inaccurate for septic tanks with long emptying intervals. Our findings suggest that emptying intervals, sludge depth, and per-capita emission factors reflecting long emptying intervals are potential parameters for accurately estimating CH_4_ emissions from septic tanks.

## Abstract 2

Shortening the emptying interval improves BOD removal efficiency and reduces the CH_4_ emission rate. CH_4_ emission estimations based on BOD removal efficiency might be inaccurate for long-emptying-interval septic tanks.

## 1 Introduction

In 2020, the population served by on-site sanitation worldwide, including septic systems and pit latrines, exceeded for the first time than that relying on sewer connections.^1^ Furthermore, since 2010, more people have reportedly been relying on septic systems than on improved latrines.^1^ In Southeast Asia, septic systems are used by a majority of the population (i.e., 90% in Vietnam,^2^ 84% in the Philippines,^3^ and 79% in Indonesia^4^). While septic systems are preferable over open defecation, they can potentially emit substantial amounts of greenhouse gases (GHGs), such as methane (CH_4_).^5−8^ Thus, assessing the GHG emissions from septic systems is crucial to achieving climate change mitigation.^9−11^ The Intergovernmental Panel on Climate Change (IPCC) approach has been widely used to estimate CH_4_ emission rates (g CH_4_/(cap·d)) based on biochemical oxygen demand (BOD).^12^ The method is based on three parameters: (i) region- or country-specific per-capita BOD (g BOD/(cap·d)), (ii) maximum CH_4_ production capacity (0.6 g CH_4_/g BOD), and (iii) CH_4_ correction factor or BOD removal efficiency of septic tanks (40–72%). Different from the IPCC, in Hanoi, BOD removal efficiencies of 10–50% have been reported.^13^ Applying the suggested BOD removal efficiency from the IPCC might lead to a considerable estimation error in CH_4_ emissions from septic systems with long emptying intervals. Therefore, the goal of this article is to investigate whether the suggested BOD removal efficiency can be used to estimate the CH_4_ emission and, if not, what alternative indicators can be used.

A septic system is usually constructed in either of the following two ways: (i) with two components, namely, a septic tank and a soil treatment unit (e.g., leach, infiltration, or drain fields) or (ii) with only a septic tank without a soil treatment unit. The septic system of type ii has to be connected to a sewerage for further treatment. However, in low- and middle-income countries, type (ii) septic tanks are frequently found and they are not always connected to sewerage but discharged to open environments.^14,15^ In this study, we focused on the septic system of type (ii) which we from here onward call septic tanks. In low- and middle-income countries in Southeast Asia, septic tanks often receive only blackwater (i.e., blackwater septic tanks), while graywater is directly discharged to a combined sewer or a drain channel.^16^

The basic function of a septic tank is to remove solids by separating settleable solids and scum of wastewater. A proportion of the settled digestible matter is stabilized in a septic tank; the untreated effluent is discharged, and a mix of stabilized and undigested solids accumulates at the bottom of the tank.^17,18^ The solids retained in septic tanks must be emptied at a proper time to maintain the functionality of the septic tank.^19−21^ The recommended emptying interval is 1–5 years.^22−24^ In this study, we use the term emptying interval to refer to the time from the latest emptying or the time after construction if there was no emptying practice. In low- and middle-income countries, long emptying intervals appear to be common; for instance, the average emptying intervals of septic tanks in Southeast Asia are 8.1 (Hanoi),^25^ 12.7 (Mandalay),^26^ and 16 (six cities in Indonesia^27^) years, indicating that the septic tanks are not operated under recommended conditions. Emptying intervals may play a significant role in septic tanks’ BOD removal efficiency, which is an important parameter for estimating CH_4_ emissions in the IPCC’s approach. However, the understanding of their complex relationships is still limited and hence merits further investigation to be able to quantify GHG emissions and develop effective mitigation strategies.

To address the goal of estimating CH_4_ emissions, we investigated the CH_4_ emission rates of septic tanks with long emptying intervals. Hanoi was selected as a study area where 84% of households use septic tanks,^28^ and the average emptying interval was reportedly 8.1 years,^25^ which is considered a long emptying interval. We collected data from 15 different septic tanks in the winter, including septage composition, influent and effluent characteristics, and CH_4_ emissions. We selected the same method to collect data on the septage composition, effluent characteristics, and CH_4_ emissions as a previous study in Hanoi in summer.^7^ This allowed us to combine the two data sets into a total of 23 different septic tanks. In the previous study, BOD removal was not measured, but we collected the influent and calculated BOD removal efficiency in the present study. We further analyzed the data with the aims to

i) assess the impact of long emptying intervals on pollutant removal efficiency and CH_4_ emission rates of septic tanks and
ii) determine the association between CH_4_ emission rates and BOD removal efficiency.

In addition, we provide a data set of the operating conditions of septic tanks (e.g., emptying intervals and sludge accumulation rate) and the characteristics of septage, influent, effluent, and CH_4_ emission rates. This data set can serve for further studies on septic tanks and other on-site sanitation in Southeast Asia and countries with similar social and climatic settings.

## 2 Materials and Methods

### 2.1 Study Area

Hanoi, the capital and second-largest city in Vietnam, located in northern Vietnam, was selected as the study area. The city covers an area of 3358.6 km^2^ with a population of 8.25 million people.^29^ Hanoi has two main seasons: summer (May to August) and winter (November to March). In summer, the weather is hot and humid with a monthly average temperature of 26–33 °C, while in winter, it is comparably cold and relatively dry with a monthly average temperature of 14–19 °C.^29,30^ In Hanoi, 94% of septic tanks receive only blackwater.^13^ These septic tanks are constructed underground usually without installing an access hole for emptying.^7^ The graywater is usually discharged directly into a drain channel or combined sewer without passing through a septic tank.^2^

### 2.2 Overview of Septic Tank Investigation

**Figure 1:**
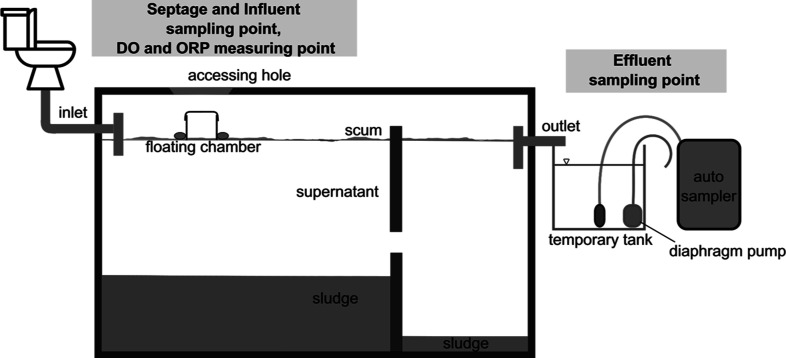
Experimental setup for septage, influent, effluent, and gas collection.

We collected data from 15 septic tanks (T1–T15) that had plans for emptying, thereby allowing us to access the septic tanks with long emptying intervals. Since none of the investigated septic tanks had any access holes for emptying, we drilled an access hole and installed a cover on top of the first compartment for emptying purposes. The septage, gas, and influent samples were collected through the access holes. The effluent samples were collected through the outlet of the septic tanks. The experimental setup is shown in Figure 1. In this study, we could locate only the first compartment of the septic tanks because this compartment is typically built directly beneath the cistern flush toilets. The second and third compartments were arranged differently from site to site and hence difficult to locate.

All 15 septic tanks were sampled in December 2019–January 2020 (winter). In addition to the data collected from septic tanks in the present study, data from 10 septic tanks (ST1–ST10) investigated by Huynh et al.^7^ in June–July 2019 (summer) was integrated into the analysis of CH_4_ emission rate in this study. Both the present study and Huynh et al.^7^ employed the same method of gas sample collection and analysis. This allowed us to investigate the seasonal variations in CH_4_ emission rates. It should be noted that T1 and T2 of the present study were the same septic tanks as ST1 and ST2 of Huynh et al.^7^ Therefore, in total, data from 23 septic tanks were used for the analysis of CH_4_ emission rates and data from 15 septic tanks in this study were used for the analysis of influent and pollutant removal efficiency.

### 2.3 Data Collection

#### 2.3.1 General Septic Tank Information

After obtaining consent to conduct the experiment from the owners of the households, we obtained information about the septic tank, including the septic tank emptying intervals, the number of toilet users, the number of septic tank compartments, and the size of the septic tanks (Table S1).

#### 2.3.2 Sample Collection

The details of the sampling schedule, including dates and frequencies of sample collection for T1–T15, are shown in Table S2.

##### 2.3.2.1 Off-Gas Measurement

Gas samples were collected in the first compartment using the floating chamber method, which was the same one used in Huynh et al.^7^ The gas collection was always carried out between 9.30 and 11.30 a.m. The time was selected together with the toilet users because the toilet was not useable during the sampling and emptying time. Additionally, this timing aligns with the data collection schedule employed by Huynh et al.^7^ The design of the floating chamber is shown in Figure S1. A floating chamber was placed on the surface of the septage through an access hole. The gas generated inside the floating chamber was collected using 24 mL syringes at *t* = 0, 10, 20, 30, and 40 min through the sampling tube connected to the chamber; this series of gas collections is referred to as one operation.

At septic tanks T1 and T2, where CH_4_ emission rates in June–July 2019 (summer) were also measured by Huynh et al.,^7^ the operation was replicated for five consecutive days to investigate seasonal variations by comparison of two data sets. The operation was carried out once for the other septic tanks (T3–T15).

##### 2.3.2.2 Septage

At the time of gas collection, sensors of oxidation–reduction potential (ORP) (9300–10D, HORIBA), dissolved oxygen (DO) (HQ30D, HACH-LDO), and electrical conductivity with water temperature (U-24, HOBO) were inserted into septage at 0.25 m below the water surface through the access holes and these parameters were measured. After gas collection, the septage was sampled through the access holes using a sludge core sampler (Figure S2A). The device consisted of a cylindrical acrylic pipe connected to a check valve at its bottom and sampling valves every 30 cm along its height. After collecting the septage with the sludge core sampler, we allowed settleable solids to be separated from the supernatant for 30 min. The separation of sludge and liquid layers was visually observed, and the sludge depth was measured, as shown in Figure S2B.

##### 2.3.2.3 Effluent

Effluent samples were collected at the outlet of the septic tanks. A temporary tank (4.5 L) was installed to store the effluent from T1 and T2 before samples were taken using an autosampler with cooling preservation by ice (3700, Teledyne ISCO). The autosampler was connected to a temporary tank to collect effluent. Effluent samples (500 mL) were collected at 2 h intervals for seven consecutive days in December 2019. A diaphragm pump was set to empty the temporary tank immediately after each 2 h collection. The composite samples of the effluent were produced daily. An average of 7 days was reported. For T3–T15, we used the grab sampling method for effluent because it was not possible to properly install the temporary tank and autosampler on the sites due to space limitations.

##### 2.3.2.4 Influent

After all other measurements were taken, the septic tanks were completely emptied by using a vacuum truck, washed with tap water, and emptied again through accessing holes. The clean, empty tank was used to store new wastewater, referred to as influent. We allowed the septic tank users to use the septic tanks for 24 h. Shortly before collecting the samples, we mixed the accumulated wastewater in the septic tank by using a long stick, and then, we collected 500 mL of the wastewater through the access hole using a bucket. For T1 and T2, we collected the influent once a day for 3 days. After each influent collection, both septic tanks were emptied and washed immediately to prepare the subsequent sample collection. One grab sample of influent was collected from each of the remaining septic tanks (T3–T15).

### 2.4 Sample Analysis

CH_4_ was analyzed for all gas samples using gas chromatography (Shimadzu GC-2014) with a flame ionization detector. The detector temperature was 200 °C with a retention time of 5.25 min. Carbon dioxide (CO_2_) was not included in this study because CO_2_ emissions from the decomposition of wastewater are biogenic and not included in the total CO_2_ equivalent estimation.^6,12^

All septage, effluent, and influent samples were kept on ice immediately after collection and transported to a refrigerator (4 °C) in the laboratory. The septage samples were analyzed for chemical oxygen demand (COD), BOD, suspended solids (SSs), and ammonium–nitrogen (NH_4_–N). The effluent and influent samples were analyzed for COD, BOD, and SS. BOD and SS analyses were performed according to standard methods.^31^ The COD and NH_4_–N were analyzed by using the HACH method (DR6000, HACH). The septage, effluent, and influent samples were duplicated for every five samples and analyzed. The presented results are averages of two analyses.

### 2.5 Data Analysis

The emission rate was calculated following the method by Diaz-Valbuena et al.^6^ In short, the CH_4_ concentration of each sample during the operation was plotted against the collection time. After verifying the linearity of the increase in CH_4_ concentration over time, the slope of the linear regression line (*y* = *mt* + *b*), *m* was obtained as the mass emission rate (g/(m^3^·d)). The CH_4_ emission rates were converted to CH_4_ emission rates per capita (g/(cap·d)) for the first compartment of the septic tanks, according to eq 1.
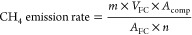
1where *V*_FC_ is the chamber volume = 0.00265 m^3^; *A*_comp_ is the area of the first compartment (m^2^); *A*_FC_ is the area covered by the floating chamber = 0.018 m^2^; and *n* is the number of septic tank users (capita). Given our goal to provide an average CH_4_ emission rate that can be used to estimate GHG emissions for septic tanks without measurements, it was important to perform an outlier test to assess the potential that a measurement leads to an overestimation of the average. Therefore, we used the interquartile range method before performing correlation analysis.^32^ We identified one extremely high CH_4_ emission rate (see Figure S3).

The removal efficiency was calculated by comparing the pollutant concentration in the influent to that in the effluent (eq 2). According to the sampling plan, we define the removal efficiency as the removed proportion of influent pollutants at the moment when the effluent and influent samples were taken. This represents the removal efficiency at the particular emptying interval, and we used it to compare to other different septic tanks.
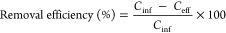
2*C*_inf_ is the influent concentration (g/m^3^), and *C*_eff_ is the effluent concentration (g/m^3^).

For a seasonal comparison of CH_4_ emissions, the results of this study (winter: December 2019–January 2020; *n* = 15) were compared with those reported by Huynh et al.^7^ (summer: June–July 2019; *n* = 10). The average ambient and liquid temperatures were 20.0 °C (17.0–24.0 °C) and 21.6 °C (19.1–24.2 °C) in winter, respectively, while they were 36.0 °C (35.0–38.0 °C) and 31.1 °C (30.1–31.7 °C) in summer.

## 3 Results and Discussion

### 3.1 General Septic Tank Conditions

The majority (83%) of the 23 septic tanks analyzed in this article had three compartments, and the others had two compartments. All of the septic tanks were constructed in a rectangular shape with bricks or reinforced concrete underneath the toilet floors. The average proportion of the first compartment was 53% of the total volume. The surveyed septic tank served 4.4 persons on average and had an average volume of 2.3 ± 1.5 m^3^ (average ± SD). Details of the septic tank design are shown in Table S3 and Figure S4. The average emptying interval was 11.7 years, ranging from 3.9 to 23.0 years. Although the average volume was lower than the minimum volume of the septic tank of at least 3.0 m^3^ of the national guidelines,^33^ the emptying intervals, tank shape, and number of users were in line with previous studies in Hanoi.^13,25,34^

The average emptying interval in Hanoi is comparably shorter than the average emptying interval of septic tanks in Thailand of 1.90 years for different types of sanitation facilities (cesspools, cement septic tanks, and plastic septic tanks).^23^ The significant difference in the emptying interval could be due to the difference in sludge accumulation rates (as discussed in Section 3.2.1) and the different designs of septic tanks. In other Southeast Asian countries, except for Thailand, the average emptying interval is also reported to exceed the recommended value of 1–5 years.^22−24^

### 3.2 Septage Accumulation and Composition against Emptying Intervals

#### 3.2.1 Sludge Depth and Sludge Accumulation Rate

Sludge depth in the first compartment of the 23 septic tanks ranged from 0.27 to 1.05 m. Seven of these septic tanks had sludge that filled the tank to more than 90% of the effective depth. A maximum depth of 1.05 m was observed in the septic tank with an emptying interval of 23.0 years; the sludge occupied 96% of the effective depth of the septic tank. Excessive accumulation of sludge resulted in a malfunction of settling solids and potentially led to the short-circuiting of raw blackwater. We conclude that these tanks cannot fulfill the basic function of a septic tank. Sludge depth was linearly correlated with the emptying interval (*R* = 0.769, *p* < 0.001), as shown in Figure S5F. More information about the sludge depth and septic tank dimensions is shown in Table S3.

The sludge accumulation rate in this study was 0.06 ± 0.05 L/(cap·d). It is comparable to 0.04 L/(cap·d) in a previous study in Hanoi^34^ and 0.07 L/(cap·d) in six cities in Indonesia,^27^ while it was high in some other countries: 0.5 L/(cap·d) in Thailand,^23^ 0.1 L/(cap·d) in France,^35^ and 0.1 L/(cap·d) in Canada.^36^ Potential factors for the difference in accumulation rate include the type of wastewater entering the septic tanks (blackwater or both black and graywater), solid and organic strength of the influent, diets, flushing water quantity, hydraulic retention time, and temperature.^17,37,38^

#### 3.2.2 Septage Composition

Detailed septage composition data are provided in Table S4. Briefly, septage COD, BOD, and SS were 16,400 ± 8,940, 13,600 ± 7,780, and 7800 ± 1860 g/m^3^, respectively. The DO concentration was 0.18 ± 0.14 g/m^3^, and the ORP value was −369 ± 97 mV. As the DO and ORP were measured at 0.25 m below the water surface and the septage depth was 0.92 m on average, these results indicate that the septage were in anaerobic conditions. An ORP of less than −330 mV is reportedly suitable for the growth of methane-forming bacteria.^39^ The favorable ORP for methanogenesis ranges from approximately −400 mV to −200 mV.^40^ The range of NH_4_–N concentration was 172–750 g N/m^3^, which did not exceed the inhibitory level for anaerobic processes of 3000 g N/m^3^.^41^ Thus, the septage DO, ORP, and NH_4_–N values potentially cause anaerobic digestion and, therefore, CH_4_ production.

As shown in Figure S5A–E, emptying intervals showed significant correlations with septage SS (*R* = 0.917, *p* < 0.001), COD (*R* = 0.914, *p* < 0.001), BOD (*R* = 0.895, *p* < 0.001), DO (*R* = −0.542, *p* = 0.005), and ORP (*R* = −0.800, *p* < 0.001). The results showed that the long emptying intervals led to the accumulation of solids and organic matter under anaerobic conditions (low DO and ORP values). Sludge depth had significant correlations with septage SS (*R* = 0.726, *p* < 0.001), COD (*R* = 0.789, *p* < 0.001), BOD (*R* = 0.775, *p* < 0.001), DO (*R* = −0.504, *p* = 0.01), and ORP (*R* = −0.583, *p* = 0.002) (Figure S6). The correlations indicate that the emptying interval and sludge depth could be used to predict the septage compositions and conditions inside septic tanks.

### 3.3 Pollutant Removal Efficiencies against Emptying Intervals

#### 3.3.1 Suspended Solids

The SS concentrations of septic tank influent and effluent were 1110 ± 321 and 142 ± 67 g/m^3^, respectively (Table S5), and the SS removal efficiency was 87.0 ± 5.8%. The effluent SS concentration was within the previously reported range of 12–733 g/m^3^ in a study in Hanoi.^13^ All of the septic tanks had an SS removal efficiency above the SS removal efficiency reported for well-functioning septic tanks (i.e., 50%).^42−44^ Possible explanations for the functionality of SS removal might be that (1) an extremely high SS concentration in the influent might allow the effective removal of a significant part of SS in septic tanks, and (2) the well-functioning second or third compartments of the tanks could help prevent solids from being carried over into the effluent.^20^ However, despite a high SS removal efficiency, it is not sufficient for septic tanks with long emptying intervals to discharge effluent with SS concentrations below the national regulation of 100 g/m^3^.^45^

**Figure 2:**
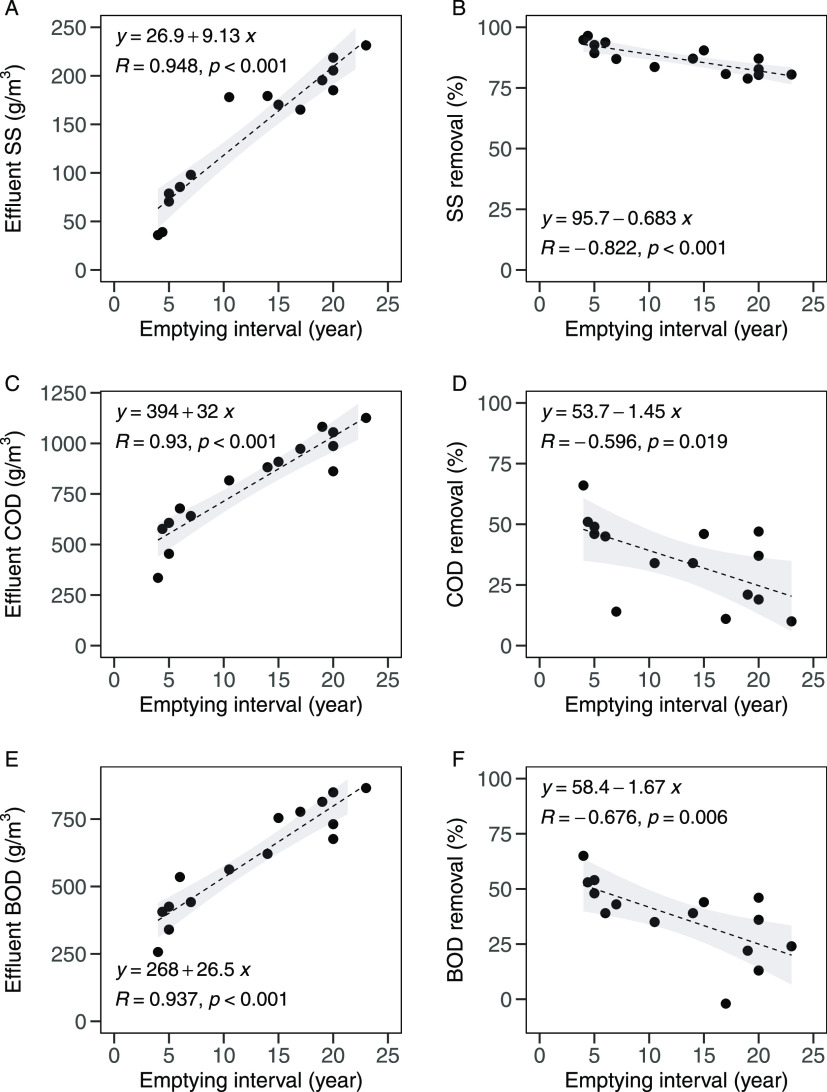
Correlations between the emptying interval and effluent SS (A); SS removal (B); effluent COD (C); COD removal (D); effluent BOD (E); and BOD removal (F) for 15 different septic tanks in Hanoi. The lines show the linear regression, and the gray zones mark the 95% confidence intervals.

Additionally, there was very strong evidence for the relationships between the emptying interval and effluent SS (*R* = 0.948, *p* < 0.001) and SS removal efficiency (*R* = −0.822, *p* < 0.001) as illustrated in Figure 2A,B, respectively. The results indicate the deterioration of the SS removal efficiency due to the long emptying interval.

#### 3.3.2 COD and BOD

The influent and effluent concentrations of COD were 1240 ± 244 and 813 ± 234 g/m^3^, respectively, and those of BOD were 937 ± 197 and 587 ± 181 g/m^3^, respectively. Subsequently, the COD and BOD removal efficiencies were calculated as 34 ± 20 and 37 ± 17%, respectively. COD and BOD concentrations of the effluent were within the ranges of a previous study in Vietnam: COD of 91–1780 g/m^3^ and BOD of 60–920 g/m^3^.^13^ The BOD removal efficiency of well-functioning septic tanks is reported as 30–50%.^42,43^ In the present study, 11 of 15 septic tanks (73%) had BOD removal efficiencies exceeding 30%. However, the effluent BOD concentrations in the 15 septic tanks were considerably higher than the national standard of 50 g/m^3^.^45^

Similar to SS, there was very strong evidence for the relationships between the emptying and effluent COD (*R* = 0.930, *p* < 0.001), BOD (*R* = 0.937, *p* < 0.001), and the removal efficiencies of COD (*R* = −0.596, *p* = 0.019) and BOD (*R* = −0.676, *p* = 0.006), indicating that prolonged use of septic tanks without emptying also deteriorated organic removal efficiencies (Figure 2C–F).

### 3.4 CH_4_ Emission

#### 3.4.1 Seasonal Variation

**Figure 3:**
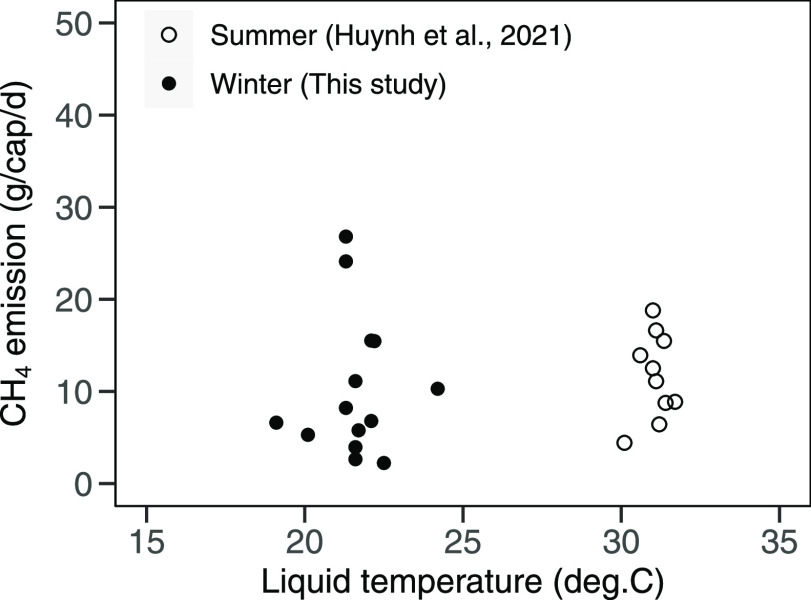
Variation of CH_4_ emission rates between the summer and winter.

The average CH_4_ emission rates in winter and summer were 10.3 ± 7.6 g/(cap·d) (range = 2.2–26.8 g/(cap·d) and 11.9 ± 4.5 g/(cap·d)^7^ (range = 4.4–18.8 g/(cap·d)), respectively. These emission rates did not differ significantly despite an average liquid temperature difference of 9.4 °C between winter (average = 21.7 °C, range = 19.1–24.2 °C) and summer (average = 31.1 °C, range = 30.1–31.7 °C). The average CH_4_ emission rate of septic tanks from both winter and summer data was 10.9 (range: 2.2–26.8) g/(cap·d). The data on CH_4_ emission rates and liquid temperature is presented in Table S7. Although CH_4_ production has been reportedly affected by temperature,^46^ there was no evidence that CH_4_ emission rates are dependent on liquid temperature among the 23 septic tanks (Figure 3). Huynh et al.^7^ reported no statistically significant correlation between CH_4_ emission rates and liquid temperature, and Diaz-Valbuena et al.^6^ also observed no clear correlation between them, while the average liquid temperature differences of Huynh et al.^7^ and Diaz-Valbuenas et al.^6^ were 1.1 and 15 °C, respectively.

We further compared the results of CH_4_ emission rates and septage composition in the winter and summer of two individual septic tanks, T1 and T2, which were the same septic tanks investigated in the two seasons (Figure S7). The CH_4_ emission rates in winter and summer of T1 were 11.1 g/(cap·d) at 21.6 °C and 11.1 g/(cap·d) at 31.1 °C, and those of T2 were 15.5 g/(cap·d) at 22.1 °C and 15.5 g/(cap·d) at 31.4 °C, respectively. The septage COD, BOD, SS, and DO concentrations and ORP were comparable between the two seasons (Table S8). CH_4_ emission rates were comparable between summer and winter, although the liquid temperature differed by 9.4 °C. Hence, the impact of temperature might have been limited, possibly because the temperature was lower than the optimal temperature of anaerobic digestion of 35–37 °C.^47^ Additionally, the impact of temperature on CH_4_ emission rates might be masked by the impact of other influential factors, such as organic accumulation (BOD and COD) and anaerobic conditions (low DO and ORP values), which are discussed in the following section.

The CH_4_ emission rates of the same septic tanks between winter and summer were in a similar range and seem to be only marginally affected by the liquid temperature difference of 9.4 °C. Hence, we integrated the data of the present study in the winter with the data of the previous study in the summer, following the same sampling and analytical methods in the summer, and analyzed the correlation of CH_4_ emission rates and other parameters of both summer and winter together. This was possible because we followed the same sampling and analytical procedures as in the previous study.

#### 3.4.2 CH_4_ Emission Rates against Emptying Intervals and Removal Efficiencies

The CH_4_ concentrations of the gases sampled from the floating chamber of the 15 septic tanks at *t* = 0, 10, 20, 30, and 40 min are listed in Table S6. We confirmed that the septic tanks produced significant concentrations of CH_4_ and the CH_4_ concentrations in the floating chamber linearly increased (*R* = 0.976–0.996), reaching 3430–22,600 g/m^3^ at *t* = 40 min.

**Figure 4:**
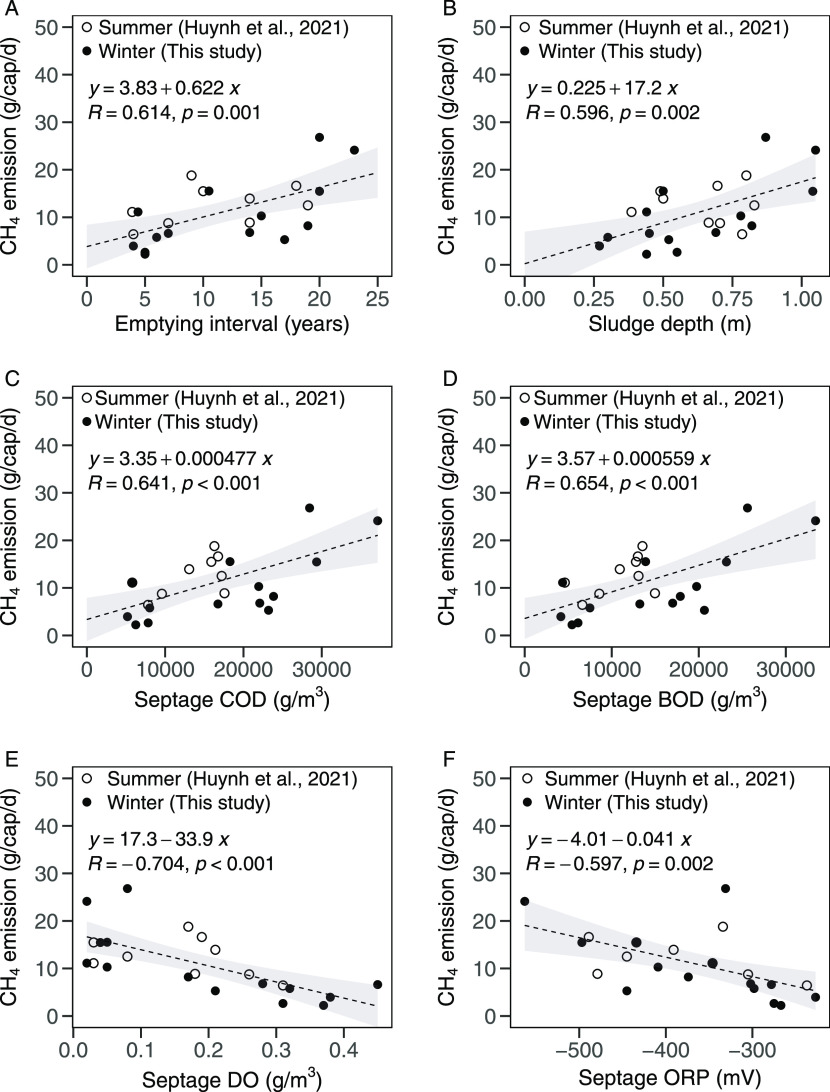
Correlations between CH_4_ emission and emptying interval (A); sludge depth (B); septage COD (C); septage BOD (D); septage DO (E); and septage ORP (F) for 15 septic tanks in Hanoi in winter (the present study) and 10 septic tanks in summer (Huynh et al.^7^). The lines show the linear regression, and the gray zones mark the 95% confidence intervals.

The CH_4_ emission rates were strongly correlated with emptying intervals (*R* = 0.614, *p* = 0.001) and sludge depth (*R* = 0.596, *p* = 0.002), as shown in Figure 4A,B. Furthermore, they were strongly correlated with the septage compositions including COD (*R* = 0.641, *p* < 0.001), BOD (*R* = 0.654, *p* < 0.001), DO (*R* = −0.704, *p* < 0.001), and ORP (*R* = −0.597, *p* = 0.002), as shown in Figure 4C–F. These correlations could be explained as follows. Emptying intervals become longer, and the sludge depth increases accordingly. Figures S5 and S6 indicate that long emptying intervals led to higher BOD and COD and lower DO and ORP in the septage, meaning organic matter accumulation under anaerobic conditions. Accordingly, CH_4_ emissions increase with increasing emptying intervals and sludge depth. A previous study also showed that higher BOD and COD concentrations and lower DO and ORP were key chemical conditions for the CH_4_ emission from septic tanks, although the study did not confirm the significant correlation between the emission and the sludge depth.^7^

**Figure 5:**
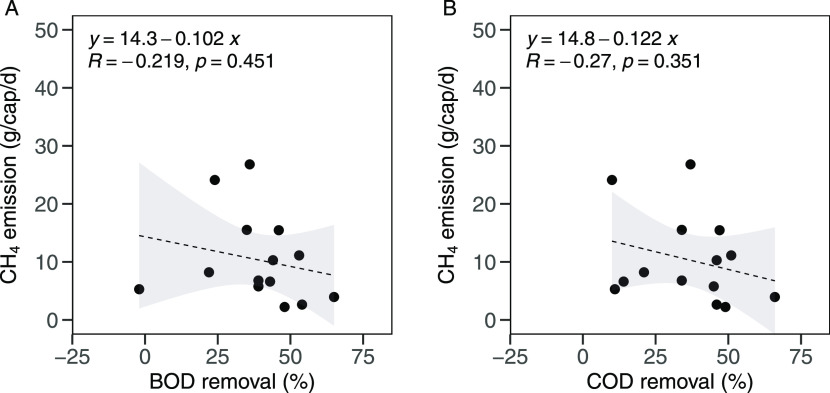
Correlations between CH_4_ emission and BOD removal (A) and COD removal (B) for 15 septic tanks in Hanoi. The lines show the linear regression, and the gray zones mark the 95% confidence intervals.

Considering the relationship between CH_4_ emission and BOD removal efficiency, one septic tank (T14) had not been emptied for 17 years and had BOD removal efficiency less than 0%, of which the CH_4_ emission rate was 5.3 g/(cap·d). The negative BOD removal rate could be due to a short circuit in the septic tank or the accumulated sludge may be carried over through the effluent. Both of these phenomena are caused by excessive sludge accumulation. Notably, the observation showed that even though the tank was not functional as a septic tank and, consequently, no longer removed BOD from the influent, CH_4_ was still emitted from the tank. The CH_4_ emission rates were plotted against the BOD and COD removal efficiencies, as shown in Figure 5. Although the correlations were not statistically confirmed, CH_4_ emission rates were negatively correlated with BOD (*R* = −0.219, *p* = 0.451) and COD (*R* = −0.270, *p* = 0.351) removal efficiencies. These indicate that the CH_4_ emission could not be estimated based on the positive correlation with BOD or COD removal efficiencies for the septic tanks with long emptying intervals.

### 3.5 Challenges and Implications to the Current CH_4_ Emission Estimation Methods

In 2010, the wastewater sector accounted for 8% of the global anthropogenic CH_4_ emissions, following enteric fermentation (28%), agriculture (20%), oil and gas (18%), and landfills (10%).^48^ From 1990 to 2005, global CH_4_ emissions from wastewater were estimated to have increased by about 35% and are predicted to increase by 28% in 2030.^49^ The major contributors to emitting CH_4_ in the wastewater sector are the low- and middle-income countries in Asia and Africa regions,^49^ where septic systems are prevalent. However, the quantification of CH_4_ emissions and thus the implementation of mitigation strategies within this sector pose significant challenges.

In the case of septic tanks, our findings indicate that shortening the emptying interval could improve pollutant removal efficiencies, including COD, BOD, and SS, thereby preventing septic tanks that have been in use for a long time and have poor functionality from discharging highly polluted effluent into the environment. For septic tanks where the effluent quality cannot meet the environmental standard, effluents must be treated by further processes such as a soil treatment unit or collected and treated at centralized wastewater treatment plants.

Furthermore, shortening the emptying interval could reduce the CH_4_ emission rates from septic tanks. For climate change mitigation and pollution control, we therefore recommend creating incentives for shortening emptying intervals of the septic tank as an efficient measure to improve the treatment efficiency of septic tanks and, at the same time, reduce GHG emissions. In the interest of fostering demand for the emptying service and supporting GHG mitigation, appropriate intervals should be considered to balance the impact on climate change and the aquatic environment with the financial burdens caused by the emptying, transportation, treatment, and disposal of emptied sludge. Additionally, even if CH_4_ emissions are mitigated from septic tanks, CH_4_ and other GHGs can potentially be emitted from other steps of the sanitation service chain, including emissions from sludge transportation, sludge treatment facilities, and disposal sites. As septic tanks are only a part of the sanitation service chain, GHG-mitigating fecal sludge management (FSM) along the entire sanitation service chain is ultimately required. Moreover, a city-wide balance might be needed to investigate if previous storage in a septic tank could lead to additional GHG emissions when compared to direct treatment in a centralized wastewater treatment plant and if, in such a case, the septic tank would better be removed to sustainably mitigate GHG emissions from urban sanitation.

Regarding CH_4_ emission estimation, the IPCC employs an approach where the CH_4_ emission rate is estimated based on a positive correlation with BOD removal efficiencies; a default value of 50% (40–72%) of the influent BOD is assumed to be removed in septic tanks, and this fraction is then converted into CH_4_.^12^ To highlight the differences, we estimated the emission rates based on IPCC and compared with our results. For the IPCC approach, we calculated CH_4_ emissions by utilizing the recommended per-capita BOD for Asia of 40 g/(cap·d) for domestic wastewater.^12^ Given that the BOD of blackwater in low- and middle-income countries accounts for 55% of the total BOD in domestic wastewater,^50^ the per-capita BOD value for blackwater would be 22 g/(cap·d). Based on the IPCC approach, accounting for maximum and minimum BOD removal efficiencies within the suggested range (i.e., 40 and 72%), the estimated CH_4_ emission rates are 5.3 and 9.5 g/(cap·d), respectively. In this present study, the average BOD removal efficiency was 37% (−2–65%) and the average CH_4_ emission rate was 10.9 (2.2–26.8) g/(cap·d). The difference between the results of our study and those of the IPCC suggests an estimation error when using BOD removal to estimate CH_4_ emission rates (Table S9).

It should be noted that in this study, 53% of 15 septic tanks could not meet the lower range of the BOD removal efficiency (40%) suggested by the IPCC and the CH_4_ emissions were only assessed in the first compartment, which accounts for 53% of the entire tank’s volume. Furthermore, the negative correlation between CH_4_ emission rates and BOD removal efficiencies demonstrated that the CH_4_ emission cannot be estimated by BOD removal efficiencies in the case of septic tanks with long emptying intervals. For better quantification, other influential parameters should be considered for the CH_4_ emission estimation. Based on our findings, emptying intervals could be a potential factor in estimating CH_4_ emissions due to their strong and significant correlation with CH_4_ emission rates and the fact that they can be obtained without conducting on-site measurements. Sludge depth could serve as a measured parameter that is obtainable with much less effort than direct CH_4_ measurements. The CH_4_ emission factor per capita obtained in the present study could also be useful data for the estimation of the city-wide emission, reflecting the reality of long emptying interval septic tanks in low- and middle-income countries. As CH_4_ emission rates were strongly correlated with the emptying intervals and emptying is a crucial component of FSM, the conditions of FSM would affect the emission from septic tanks in the city. Accordingly, the GHG emission estimation should include the factor of the FSM. Hence, the distribution of emptying intervals and/or sludge depth in the city would be a key factor to reflect the effect of FSM on the city-wide estimation.

In conclusion, this study examined how emptying intervals affect the septic tank removal efficiency and CH_4_. The main findings are as follows.

1. Longer emptying intervals significantly reduced the pollutant removal efficiencies, while they increased the CH_4_ emission rate from the septic tanks.
2. The negative correlation between BOD removal efficiency and CH_4_ emission rates signals that the CH_4_ emission estimation based on BOD removal efficiency might not reflect a realistic emission for long emptying interval septic tanks. For better quantification, we suggest using alternative factors for CH_4_ emission, including emptying intervals, sludge depth, and CH_4_ emission factors per capita reflecting long emptying intervals.

The following limitations of this study should be noted. The present study investigated only the first compartment of the septic tanks. Further studies are required to investigate potential CH_4_ emissions from other compartments. An appropriate emptying strategy should be explored to balance the financial cost and environmental impacts, including water pollution and climate change. Nevertheless, the findings of the present study and the data set of septic tanks with long emptying intervals are crucial for mitigating GHG emissions from septic tanks and exploring strategic septic tank usage/removal and FSM in low- and middle-income countries.

## Supporting Information Available

Septic tank information from households; sampling schedule for effluent, influent, septage, and gas collection; general septic tank conditions; septage compositions; influent and effluent compositions and septic tank efficiency; CH_4_ concentrations in the floating chamber; CH_4_ emission rates and liquid and ambient temperature; septage compositions between summer and winter; CH_4_ emission rates from IPCC method and direct measurement; floating chamber design; a sludge core sampler design; box plot of CH_4_ emission rates; plans of septic tanks; correlations between emptying intervals and septage compositions; correlations between sludge depth and septage compositions; septage composition and CH_4_ emission rates from summer and winter of T1 and T2 (PDF)

## Abbreviations

BOD: biochemical oxygen demand
CH_4_: methane
COD: chemical oxygen demand
DO: dissolved oxygen
FSM: fecal sludge management
GHG: greenhouse gas
IPCC: Intergovernmental Panel on Climate Change
NH_4_–N: ammonium–nitrogen
ORP: oxidation–reduction potential
SS: suspended solids
