# Supplementary material for: Challenges to Accurate Estimation of Methane Emission from Septic Tanks with Long Emptying Intervals
Source: Environ Sci Technol. 2023 Oct 19;57(43):16575–84. doi: 10.1021/acs.est.3c05724 (PMC10621000; doi:10.1021/acs.est.3c05724)
Supplement: Supplementary file 1 — es3c05724_si_001.pdf [file es3c05724_si_001.pdf]

Supporting information

Challenges to accurate estimation of methane emission from septic tanks with long emptying intervals

Jakpong Moonkawin[a], Loi T. Huynh[b], Mariane Y. Schneider[c, d, e], Shigeo Fujii[a], Shinya Echigo[a], Lien P. H. Nguyen[f], Thu-Huong T. Hoang[f], Hai T. Huynh[f], Hidenori Harada[g]*

[a] Graduate School of Global Environmental Studies, Kyoto University, Kyoto 606-8501, Japan

[b] Faculty of Environment, School of Technology, Van Lang University, Ho Chi Minh City 70000, Vietnam

[c] Next Generation Artificial Intelligence Research Center & School of Information Science and Technology, The University of Tokyo, 113-8656 Tokyo, Japan

[d] BIOMATH, Department of Data Analysis and Mathematical Modelling, Ghent University, Coupure Links 653, Ghent 9000, Belgium

[e] Centre for Advanced Process Technology for Urban Resource Recovery (CAPTURE), Frieda Saeysstraat 1, Gent 9000, Belgium

[f] School of Chemistry and Life Science, Hanoi University of Science and Technology, Hanoi 10000, Vietnam

[g] Graduate School of Asian and African Area Studies, Kyoto University, Kyoto 606-8501, Japan

* Corresponding author: Hidenori Harada (Email: harada.hidenori.8v@kyoto-u.ac.jp)

Number of pages: 13

Number of tables: 9

Number of figures: 7

Table S1 Septic tank information from households

No.	Information
1	Emptying interval (year)
2	Number of toilet users (person)
3	Number of septic tank compartments
4	Entire tank dimension
	Width (m)
	Length (m)

* All households' owners gave consent orally. No personal and sensitive data were collected in this study.

Table S2 Sampling schedule for effluent, influent, septage and gas collection

Septic tank	Samples	December 2019																										January 2020								
		6	7	8	9	10	11	12	13	14	15	16	17	18	19	20	21	22	23	24	25	26	27	28	29	30	31	1	2	3	4	5	6	7	8	9
T1	eff (•), inf (×)	•	•	•	•	•	•	•				×	×	×																						
	gas (□)						□	□	□	□	□																									
	sludge (◊)										◊																									
T2	eff (•), inf (×)											•	•	•	•	•	•	•	×	×	×															
	gas (□)													□	□	□	□	□																		
	sludge (◊)																	◊																		
T3	eff (•), inf (×)																•	×																		
	gas (□)																□																			
	sludge (◊)																◊																			
T4	eff (•), inf (×)																					•	×													
	gas (□)																					□														
	sludge (◊)																					◊														
T5	eff (•), inf (×)																								•	×										
	gas (□)																								□											
	sludge (◊)																								◊											
T6	eff (•), inf (×)																									•	×									
	gas (□)																									□										
	sludge (◊)																									◊										
T7	eff (•), inf (×)																												•	×						
	gas (□)																												□							
	sludge (◊)																												◊							
T8	eff (•), inf (×)																												•	×						
	gas (□)																												□							
	sludge (◊)																												◊							
T9	eff (•), inf (×)																													•	×					
	gas (□)																													□						
	sludge (◊)																													◊						
T10	eff (•), inf (×)																													•	×					
	gas (□)																													□						
	sludge (◊)																													◊						
T11	eff (•), inf (×)																													•	×					
	gas (□)																													□						
	sludge (◊)																													◊						
T12	eff (•), inf (×)																														•	×				
	gas (□)																														□					
	sludge (◊)																														◊					
T13	eff (•), inf (×)																														•	×				
	gas (□)																														□					
	sludge (◊)																														◊					
T14	eff (•), inf (×)																														•	×				
	gas (□)																														□					
	sludge (◊)																														◊					
T15	eff (•), inf (×)																																		•	×
	gas (□)																																		□	
	sludge (◊)																																		◊	

Table S3 General septic tanks conditions

Septic tank	Emptying interval (years)	Toilet user (person)	Sludge depth (m)	Sludge accumulation rate (L/(cap·d)	No. of compartment	Entire septic tank[1]		First compartment[2]			First/Entire tank
						Width (m)	Length (m)	Width (m)	Length (m)	Depth (m)	
T1	4.4	5	0.44	0.12	3	3	3	1.45	1.45	0.87	23%
T2	10.5	5	0.50	0.04	3	1.5	1.5	0.98	1.4	0.70	61%
T3	7	5	0.45	0.03	3	1.4	1.2	0.7	1.4	0.90	58%
T4	23	4	1.05	0.04	3	1.9	1.2	0.65	1.85	1.09	53%
T5	20	4	1.04	0.05	2	1.9	1.7	0.7	1.84	1.11	40%
T6	19	5	0.82	0.03	3	1.5	1.3	0.97	1.21	0.87	60%
T7	20	2	0.87	0.07	3	1.5	1.4	0.88	1.42	0.94	60%
T8	5	4	0.55	0.05	2	1.2	1.1	0.71	1.01	0.75	54%
T9	14	4	0.69	0.02	2	1.2	1	0.69	0.92	0.81	53%
T10	20	4	0.90	0.10	3	2.3	1.9	1.37	2.25	1.12	71%
T11	5	5	0.44	0.03	3	1.2	0.9	0.71	0.83	0.95	55%
T12	4	4	0.27	0.06	3	1.6	1.4	0.87	1.52	0.89	59%
T13	6	5	0.30	0.03	3	1.3	1.3	0.72	1.26	0.86	54%
T14	17	5	0.52	0.01	3	1.2	1	0.68	0.92	0.78	52%
T15	15	4	0.78	0.03	3	1.7	1.2	0.81	1.15	1.12	46%
ST1	3.9	5	0.39	0.11	3	2	1.5	1.45	1.45	0.87	70%
ST2	10	5	0.49	0.04	3	1.5	1.5	1.4	0.98	0.70	61%
ST3	14	4	0.66	0.04	3	2	1.5	1.45	0.95	0.72	46%
ST4	7	4	0.71	0.17	3	2.3	1.5	1.67	1.45	0.94	70%
ST5	18	3	0.70	0.05	3	1.5	1.5	1.495	1.01	1.09	67%
ST6	4	4	0.79	0.17	2	1.5	1.3	1.285	0.995	0.84	66%
ST7	9	3	0.80	0.15	3	1.7	1.5	1.51	1.2	1.18	71%
ST8	19	6	0.83	0.03	3	1.5	1.5	1.51	0.995	0.88	67%
ST9	14	5	0.50	0.04	3	2	1.5	1.51	1.495	1.00	75%
ST10	4	6	0.30	0.03	3	1.2	1.2	1.185	0.69	1.00	57%
**Average**	**11.7**	**4.4**	**0.63**	**0.06**	**2.8**	**1.66**	**1.42**	**1.09**	**1.27**	**0.92**	**58%**
**SD**	**6.5**	**0.9**	**0.23**	**0.05**	**0.4**	**0.43**	**0.40**	**0.36**	**0.36**	**0.14**	**11%**

[1]ST1-ST10 are obtained from Huynh et al. (2021)

[2]The data was from the interview of houses' owners

[3]The data was from direct measurement

Table S4 Septage compositions

Septic tank[1]	Parameter					
	**DO (g/m^3)**	**ORP (-mV)[2]**	**COD (g/m^3)**	**BOD (g/m^3)**	**SS (g/m^3)**	**NH_4-N (g-N/m^3)**
T1	0.02	-346	5800	4350	5472	242
T2	0.05	-434	18280	13893	7944	290
T3	0.45	-278	16740	13225	6800	353
T4	0.02	-563	37135	33422	10860	230
T5	0.04	-497	29340	23179	9822	700
T6	0.17	-374	23840	17880	8166	315
T7	0.08	-331	28441	25597	9382	620
T8	0.31	-275	7830	6123	5216	570
T9	0.28	-302	22080	17002	8854	500
T10	0.02	-501	29592	25153	9594	360
T11	0.37	-267	6245	5434	5307	225
T12	0.38	-227	5190	4152	4855	235
T13	0.32	-298	8024	7490	5682	425
T14	0.21	-445	23200	20648	9637	750
T15	0.05	-409	21945	19751	8422	615
ST1	0.03	-346	5800	4606.5	5895	258
ST2	0.03	-434	15892	12781.5	7805	278
ST3	0.18	-479	17576	14964	9264	478
ST4	0.26	-305	9584	8571	8830	584
ST5	0.19	-489	16758	13001	9870	533
ST6	0.31	-237	7820	6621	6020	175
ST7	0.17	-334	16286	13529	6788	532
ST8	0.08	-445	17220	13076	9934	451
ST9	0.21	-391	13068	10924	9067	440
ST10	0.34	-230	6250	5013	5573	172
**Average**	**0.18**	**-369**	**16397**	**13615**	**7802**	**413**
**SD**	**0.14**	**96**	**8941**	**7782**	**1861**	**170**

[1]ST1-ST10 are obtained from Huynh et al. (2021)

[1]ORP_{SHE} (mV) = ORP + 206 − 0.7(t − 25), where t is the temperature (°C)

Table S5 Influent and effluent compositions and septic tank efficiency

Septic tank	Influent(g/m^3)*					Effluent(g/m^3)*					Removal rate**			
	COD	BOD	SS	NH_4-N	pH	COD	BOD	SS	NH4-N	pH	COD	BOD	SS	NH_4-N
T1	1,222	930	1,099	283	7.57	602	440	40	268	7.5	51%	53%	96%	5%
T2	1,236	872	1,087	295	7.81	817	563	178	299	7.8	34%	35%	84%	-1%
T3	971	777	750	564	7.71	831	442	98	297	8	14%	43%	87%	47%
T4	1,024	768	1,188	286	8.4	1,126	582	231	263	7.4	10%	24%	81%	8%
T5	1,631	1,256	1,113	268	7.76	862	676	182	319	7.7	47%	46%	84%	-19%
T6	1,368	1,040	924	233	7.74	1,082	814	195	247	8	21%	22%	79%	-6%
T7	1,574	1,149	1,588	631	7.56	987	731	206	249	7.7	37%	36%	87%	61%
T8	1,128	914	1,077	312	8.21	607	425	79	284	7.3	46%	54%	93%	9%
T9	1,327	1,022	1,387	270	7.81	882	621	179	325	7.9	34%	39%	87%	-20%
T10	1,299	974	1,071	266	7.94	1,055	849	185	343	7.5	19%	13%	83%	-29%
T11	887	648	662	435	8.75	454	340	71	292	7.4	49%	48%	89%	33%
T12	991	733	690	269	8.55	335	257	36	275	7.4	66%	65%	95%	-2%
T13	1,229	873	1,375	336	7.62	678	535	86	303	7.7	45%	39%	94%	10%
T14	1,093	765	857	503	7.87	973	777	165	320	7.7	11%	-2%	81%	36%
T15	1,693	1,337	1,785	431	8.45	909	754	170	290	7.8	46%	44%	90%	33%
**Average**	**1245**	**937**	**1110**	**359**	**8.0**	**813**	**587**	**140**	**292**	**7.7**	**34%**	**37%**	**87%**	**11%**
**SD**	**245**	**197**	**321**	**124**	**0.4**	**234**	**181**	**64**	**28**	**0.2**	**20%**	**17%**	**6%**	**26%**

* The units for all parameters are presented in mg/L except pH

** Average of removal rates were calculated based on both positive and negative data

Table S6 CH_4 concentrations in floating chamber at different time

Septic tank	CH_4 concentration (g/m^3)				
	t=0	t=10	t=20	t=30	t=40
T1*	8	2,209	4,140	5984	7,375
T2*	9	4,963	8,337	13,366	15,665
T3	5	2,074	5,544	7,313	9,083
T4	6	7,645	12,881	18,629	22,571
T5	7	3,016	7,632	10,227	13,243
T6	7	3,094	6,481	7,648	9,955
T7	2	3,563	7,524	9,885	11,846
T8	3	1,354	2,507	3,255	4,199
T9	5	2,583	6,059	9,563	11,514
T10	5	5,438	8,261	15,239	16,317
T11	4	2,394	2,785	4,575	5,566
T12	4	1,531	2,088	3,045	3,433
T13	10	2,462	3,514	5,566	9,618
T14	6	3,357	6,713	9,966	11,420
T15	5	3,213	5,061	9,390	12,517
**Average**	6	3,260	5,968	8,910	10,955
**SD**	2	1,648	2,831	4,374	4,966

* The concentrations are averages from five operations

Table S7 CH_4 emission rates, liquid and ambient temperatures

Septic tank[1]	CH_4 emission (g/(cap·d))	liquid temperature (°C)	Ambient temperature (°C)
T1	11.12	21.6	19
T2	15.52	22.1	19
T3	6.61	19.1	20
T4	24.12	21.3	21
T5	15.46	22.2	23
T6	8.21	21.3	24
T7	26.81	21.3	21
T8	2.64	21.6	17
T9	6.79	22.1	18
T10	46.38	23.6	18
T11	2.23	22.5	22
T12	3.95	21.6	21
T13	5.78	21.7	18
T14	5.29	20.1	19
T15	10.29	24.2	24
ST1	11.11	31.1	37
ST2	15.48	31.4	35
ST3	8.87	31.7	36
ST4	8.76	31.4	38
ST5	16.62	31.1	38
ST6	6.43	31.2	35
ST7	18.79	31.0	35
ST8	12.51	31.0	35
ST9	13.93	30.6	36
ST10	4.42	30.1	38

[1]ST1-ST10 are obtained from Huynh et al. (2021)

Table S8 Septage composition between summer and winter

Parameter	T1		T2	
	Summer*	Winter**	Summer*	Winter**
COD (g/m^3)	5,800	5,800	15,892	18,280
BOD (g/m^3)	4,607	4,350	12,782	13,893
SS (g/m^3)	5,895	5,472	7,805	7,944
DO (g/m^3)	0.03	0.02	0.03	0.05
ORP (-mV)	-346	-346	-434	-434

* Summer data are obtained from Huynh et al. (2021).

** Winter data are obtained from this present study.

Table S9 CH_4 emissions rates from IPCC method and direct measurement.

Method	BOD removal efficiency or Methane correction factor (MCF)	B_0 (g CH_4/g BOD)	Per-capita BOD (BOD) (g/(cap·d))	Equation	CH_4 emissions (g/(cap·d))
IPCC	40% (min.)	0.6	22	$CH_4 emission = B_0 \cdot MCF \cdot BOD$	5.3
	50% (default)	0.6	22		6.6
	72% (max.)	0.6	22		9.5
This study	Estimation based on direct CH_4 off-gas measurement from the first compartment of 23 blackwater septic tanks in Hanoi with long emptying intervals				10.9 ± 6.46

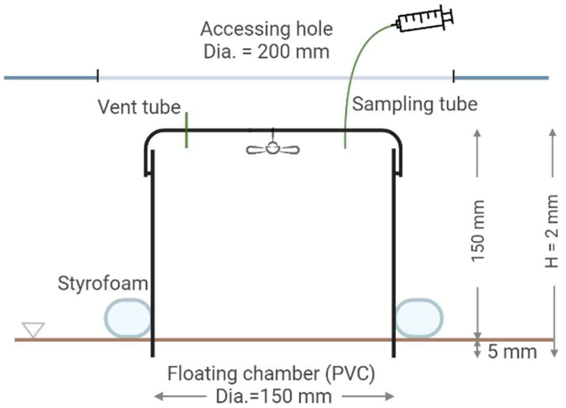

Figure S1 Floating chamber design

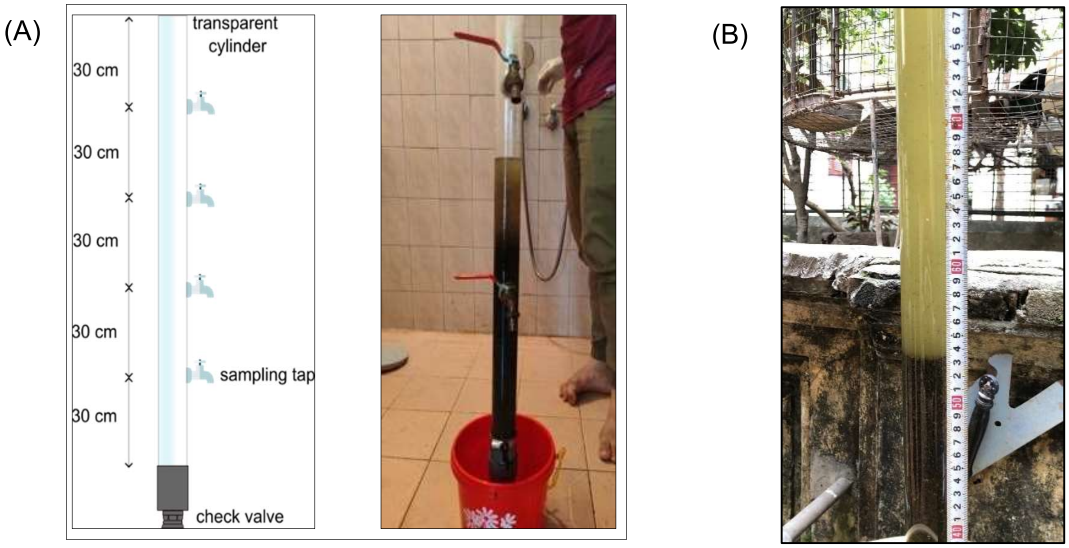

Figure S2 A sludge core sampler consisting of transparent tubes with a check valve at the bottom and sampling valves every 30 cm (A), Sludge and supernatant separation after 30 mins of collection (B)

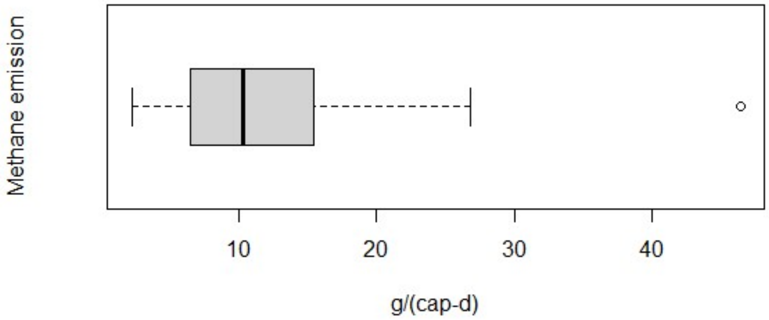

Figure S3. Box plot of CH_4 emission rates

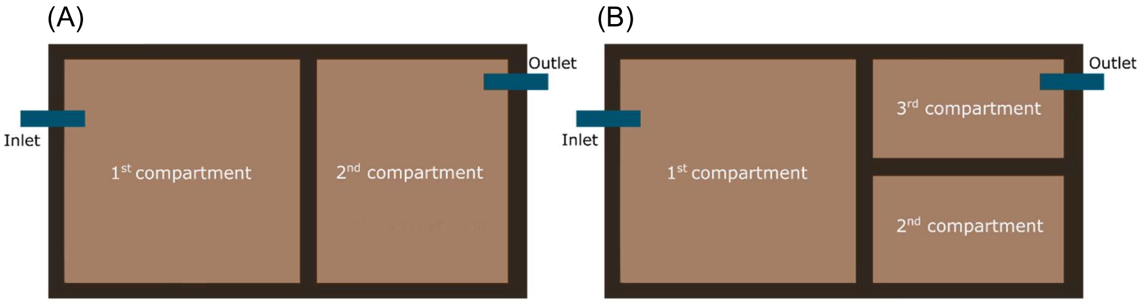

Figure S4 Plans of septic tank with two compartments (A), the plan of septic tank with three compartments (B)

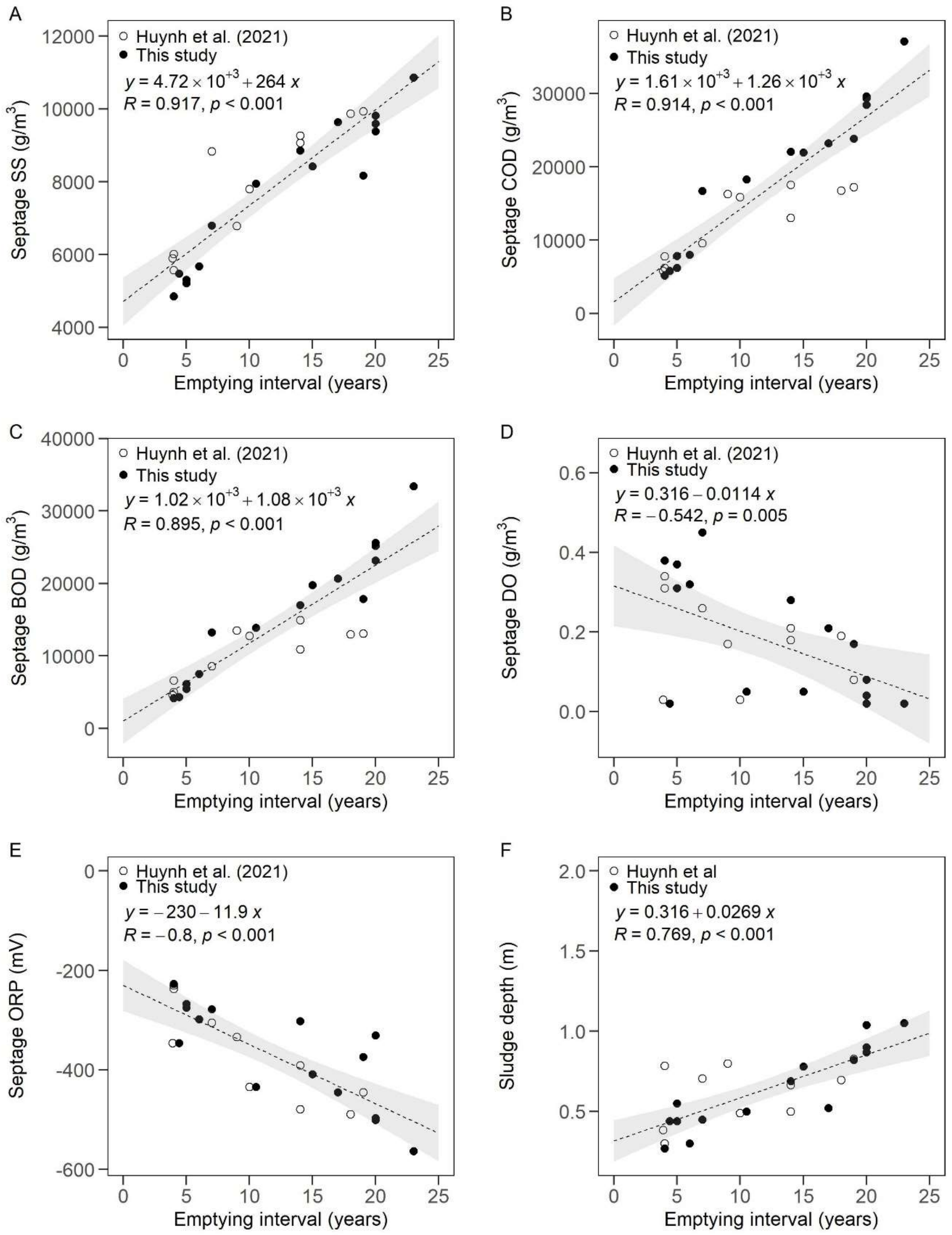

Figure S5 Correlations between emptying intervals and septage SS (A); septage COD (B); septage BOD (C); septage DO (D) and septage ORP (E), and sludge depth (F) for 25 septic tanks in Hanoi. The lines show the linear regression and the gray zones mark the 95% confidence intervals.

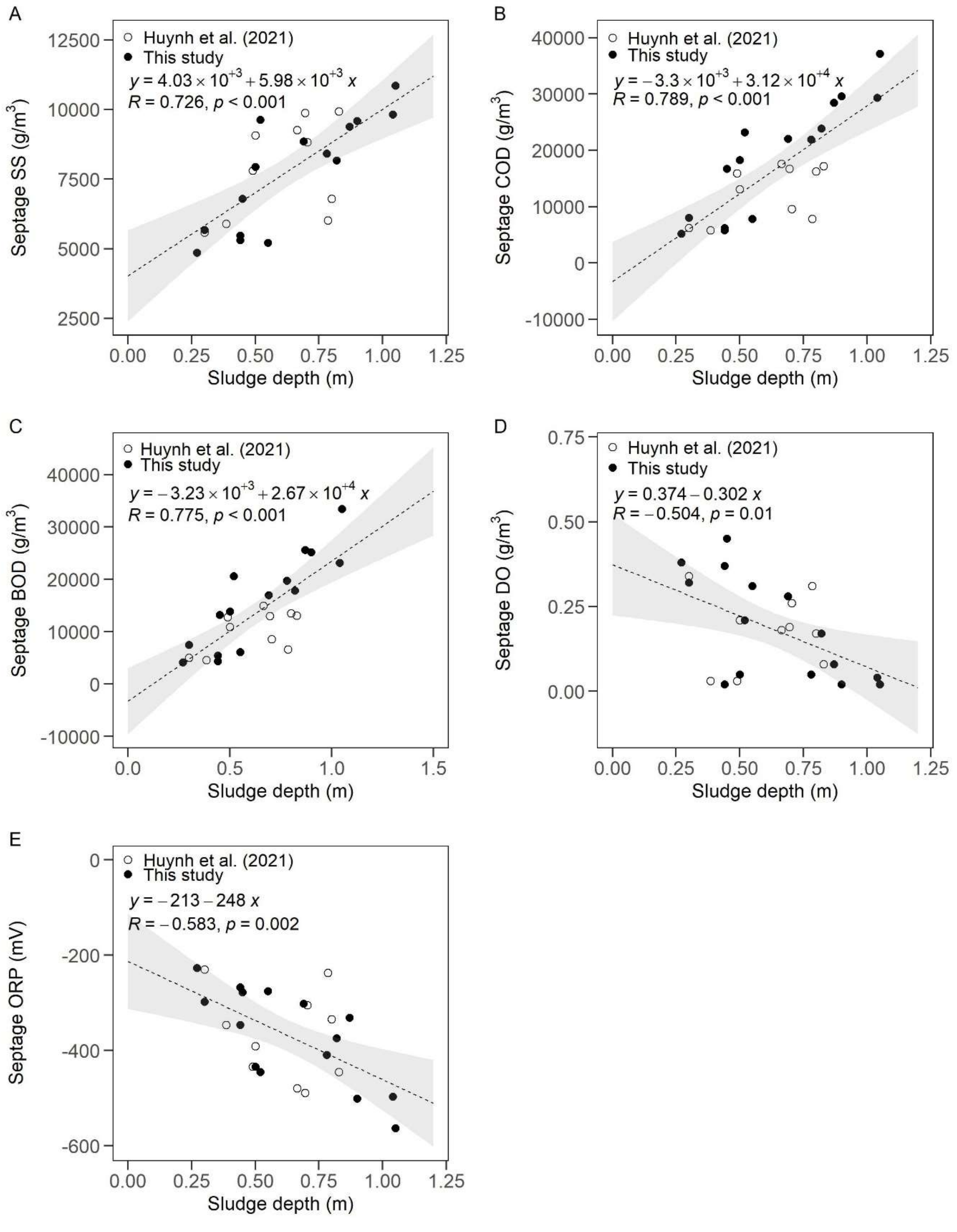

Figure S6 Correlations between sludge depth and septage SS (A); septage COD (B); septage BOD (C); septage DO (D) and septage ORP (E) for 25 septic tanks in Hanoi. The lines show the linear regression and the gray zones mark the 95% confidence intervals.

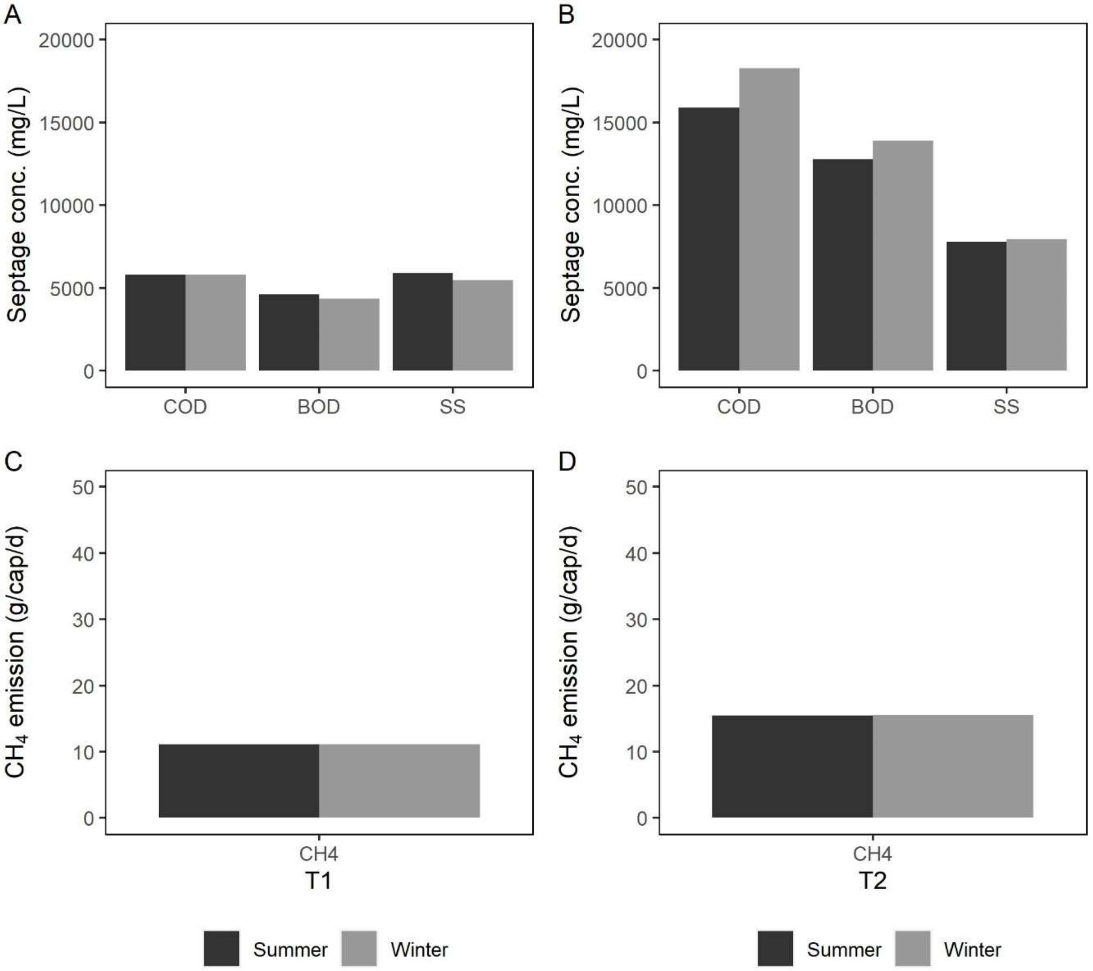

Figure S7 Septage compositions and CH_4 emission rate from summer and winter of septic tank T1 (A, C)and T2 (B, D)
